# Posterior Reversible Encephalopathy Syndrome in a Patient With Intrahepatic Cholangiocarcinoma: Converging Effects of Acute Hypertension and Cisplatin Therapy

**DOI:** 10.7759/cureus.101481

**Published:** 2026-01-13

**Authors:** George K Annan, Jefferson Awah, David A Terrero, Oladipo Odeyinka, Brooklyne M Makaya

**Affiliations:** 1 Internal Medicine, Piedmont Athens Regional Medical Center, Athens, USA

**Keywords:** acute hypertension, chemotherapy-associated neurotoxicity, cisplatin, endothelial dysfunction, intrahepatic cholangiocarcinoma, magnetic resonance imaging, oncology-related neurologic complications, posterior reversible encephalopathy syndrome (pres), pres, seizures

## Abstract

Posterior reversible encephalopathy syndrome (PRES) is a neurotoxic state characterized by seizures, headache, altered mental status, and characteristic posterior-predominant vasogenic edema on neuroimaging. It is frequently associated with acute hypertension, endothelial dysfunction, and exposure to cytotoxic or immunosuppressive agents. We report a case of PRES in a patient with stage IIIB intrahepatic cholangiocarcinoma receiving cisplatin-based chemotherapy who presented with new-onset generalized tonic-clonic seizures in the setting of newly diagnosed acute hypertension. Magnetic resonance imaging demonstrated posterior-predominant subcortical fluid-attenuated inversion recovery (FLAIR) hyperintensities consistent with PRES. The patient improved with strict blood pressure control, antiepileptic therapy, and supportive care. This case illustrates the contribution of coexisting risk factors, including acute hypertension and cisplatin exposure, to the development of PRES. It also highlights the need for broadened differential diagnoses, particularly in oncology patients in whom neurologic symptoms may initially be attributed to alternative etiologies.

## Introduction

Posterior reversible encephalopathy syndrome (PRES) is a neurotoxic condition characterized by acute neurologic symptoms and reversible vasogenic edema predominantly affecting the posterior cerebral circulation. It is characterized by headache, encephalopathy, seizures, and visual disturbances [1,2].

The incidence of PRES in the general population has been difficult to establish. In one study, the age-standardized and sex-standardized incidence of PRES was approximately 2.7 cases per 100,000 persons per year [3]. While initially described in association with hypertensive emergencies, PRES is now recognized to occur in diverse clinical contexts, including malignancy, renal dysfunction, autoimmune disease, and exposure to cytotoxic or immunosuppressive therapies [1,4].

Rather than resulting from a single insult, PRES is increasingly understood as the consequence of converging pathophysiologic mechanisms with endothelial dysfunction as the final common pathway, regardless of the initiating trigger [2]. Acute severe hypertension is presumed to damage the blood-brain barrier directly, resulting in vasogenic edema that is typically most severe in white matter [1,5]. Platinum-based chemotherapeutic agents, such as cisplatin, have been implicated in PRES through direct endothelial toxicity and disruption of the blood-brain barrier [1,6]. In oncology patients, these overlapping risk factors often coexist and may increase vulnerability to PRES. Early recognition is essential, as prompt intervention typically results in clinical and radiographic reversibility.

## Case presentation

A 61-year-old woman with stage IIIB intrahepatic cholangiocarcinoma with intrahepatic metastases presented to the emergency department with acute severe headache, followed by collapse and witnessed generalized tonic-clonic seizure activity at home. The seizure lasted less than five minutes, aborted spontaneously without any medication, and was followed by postictal confusion. During emergency medical services transport, she experienced a second generalized seizure and received intravenous midazolam.

Her oncologic treatment regimen consisted of cisplatin, gemcitabine, and durvalumab, with the most recent chemotherapy administered one day before presentation. She received cisplatin of 40 mg, gemcitabine of 1,600 mg, and durvalumab of 1,500 mg as part of her chemotherapy regimen. She reported several days of poor oral intake, progressive weakness, and nausea, but denied prior seizures, visual changes, fever, or infectious symptoms.

On presentation, vital signs revealed elevated blood pressure, with systolic readings exceeding 170 mmHg. The physical examination revealed somnolence, but the patient was easily rousable and did not exhibit any focal neurologic deficits. Initial laboratory evaluation showed leukocytosis and lactic acidosis attributed to seizure activity, which rapidly resolved. Liver function testing revealed mild elevation of transaminases but preserved synthetic function, as indicated by normal bilirubin and international normalized ratio. Serum ammonia was mildly elevated.

Non-contrast computed tomography of the head demonstrated no acute intracranial abnormality, and an electroencephalogram (EEG) was unremarkable. Given her malignancy, intracranial metastasis was initially considered. Hepatic encephalopathy was also briefly entertained early in the course due to underlying liver metastasis and elevated ammonia; however, the absence of acute liver failure, preserved synthetic function, and lack of clinical correlation made this diagnosis unlikely.

**Figure 1:**
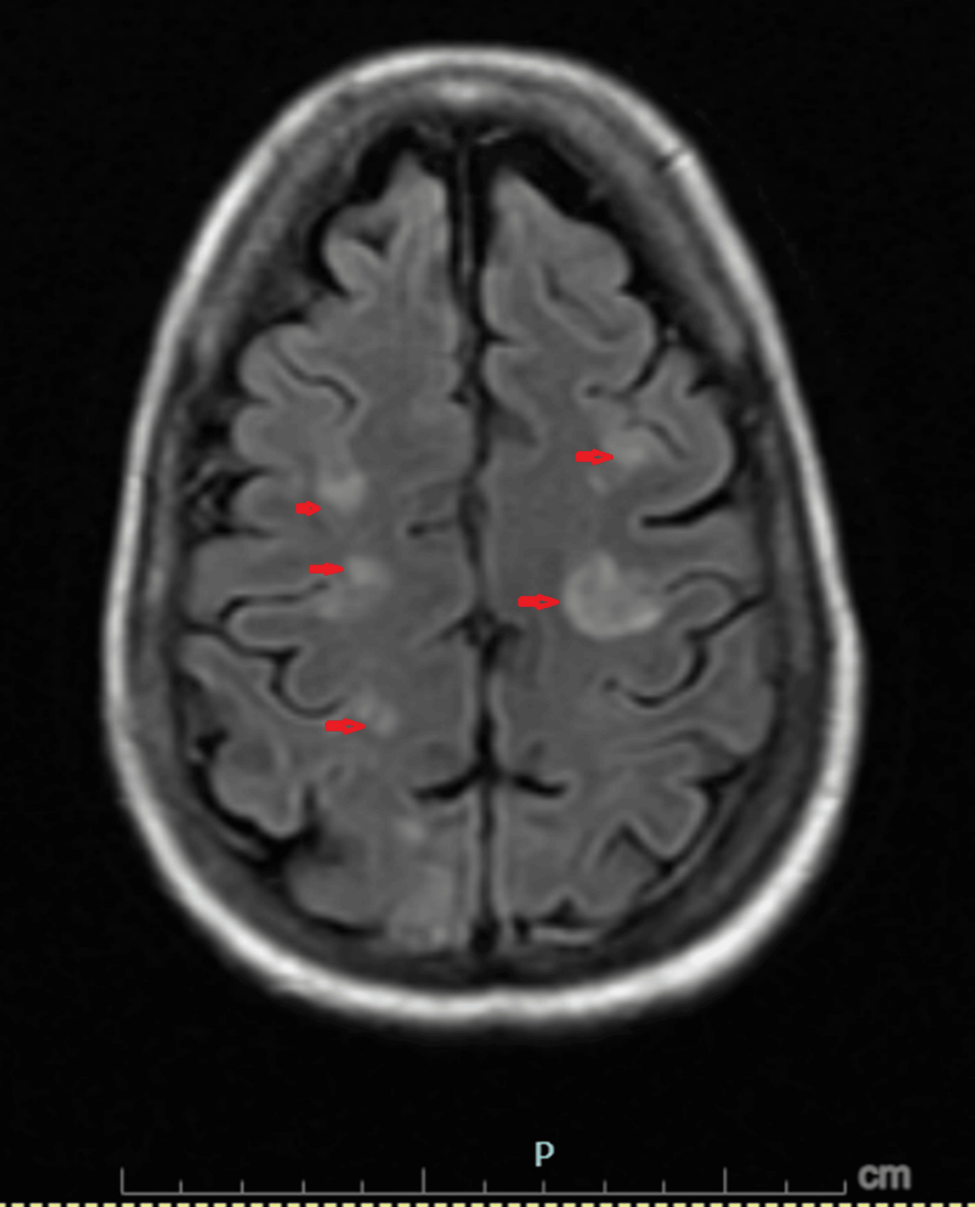
Cerebral involvement on brain MRI Magnetic resonance imaging (MRI) of the brain with fluid-attenuated inversion recovery (FLAIR) sequences demonstrating symmetric subcortical hyperintensities (red arrows) involving the bilateral cerebral hemispheres. There is no associated mass effect, diffusion restriction, or abnormal enhancement.

**Figure 2:**
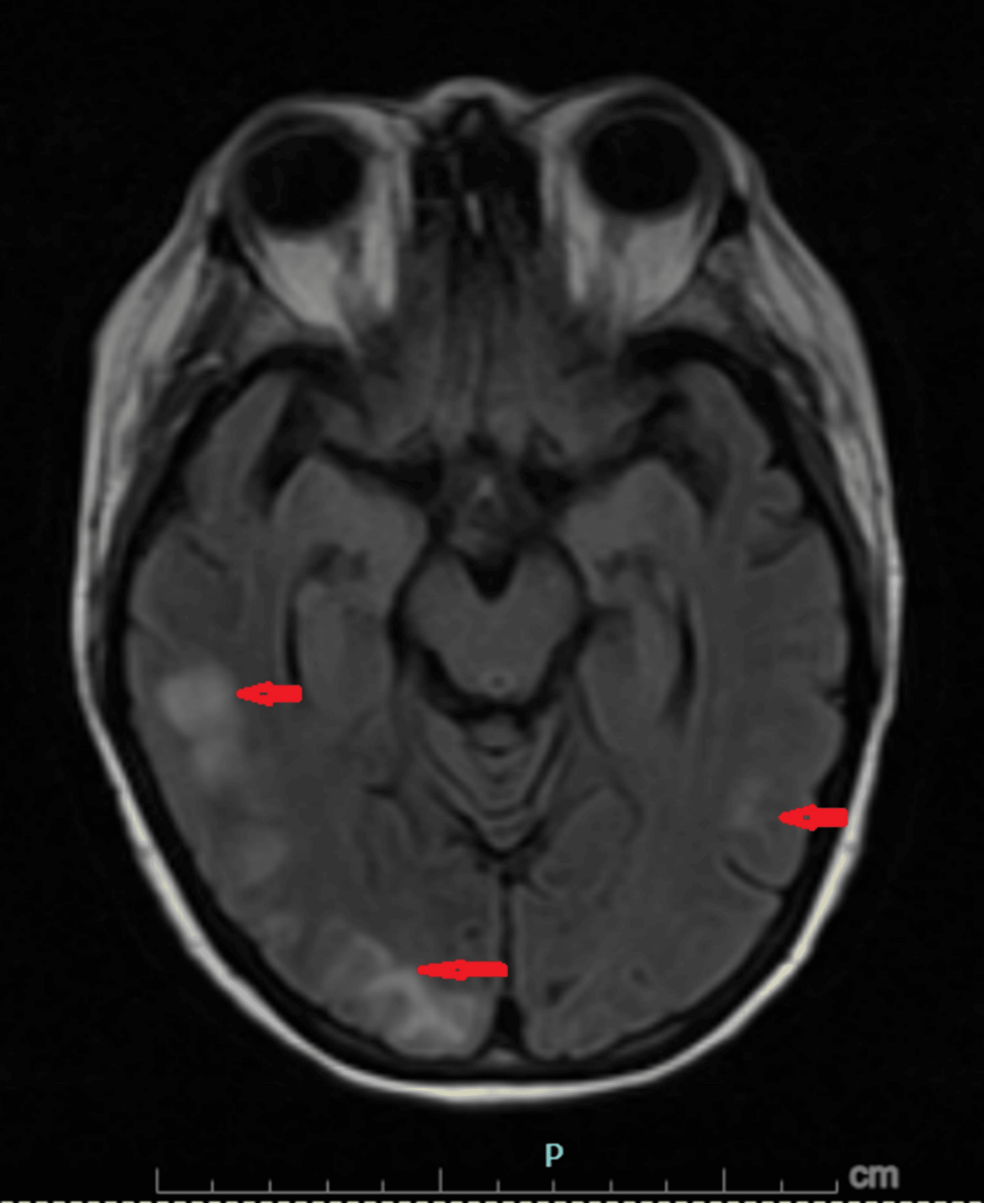
Posterior cerebral involvement on brain MRI Magnetic resonance imaging (MRI) of the brain with fluid-attenuated inversion recovery (FLAIR) sequences demonstrating subcortical hyperintensities (red arrows) involving the bilateral cerebral hemispheres with posterior predominance. There is no associated mass effect, diffusion restriction, or abnormal enhancement. These findings are characteristic of posterior reversible encephalopathy syndrome (PRES).

**Figure 3:**
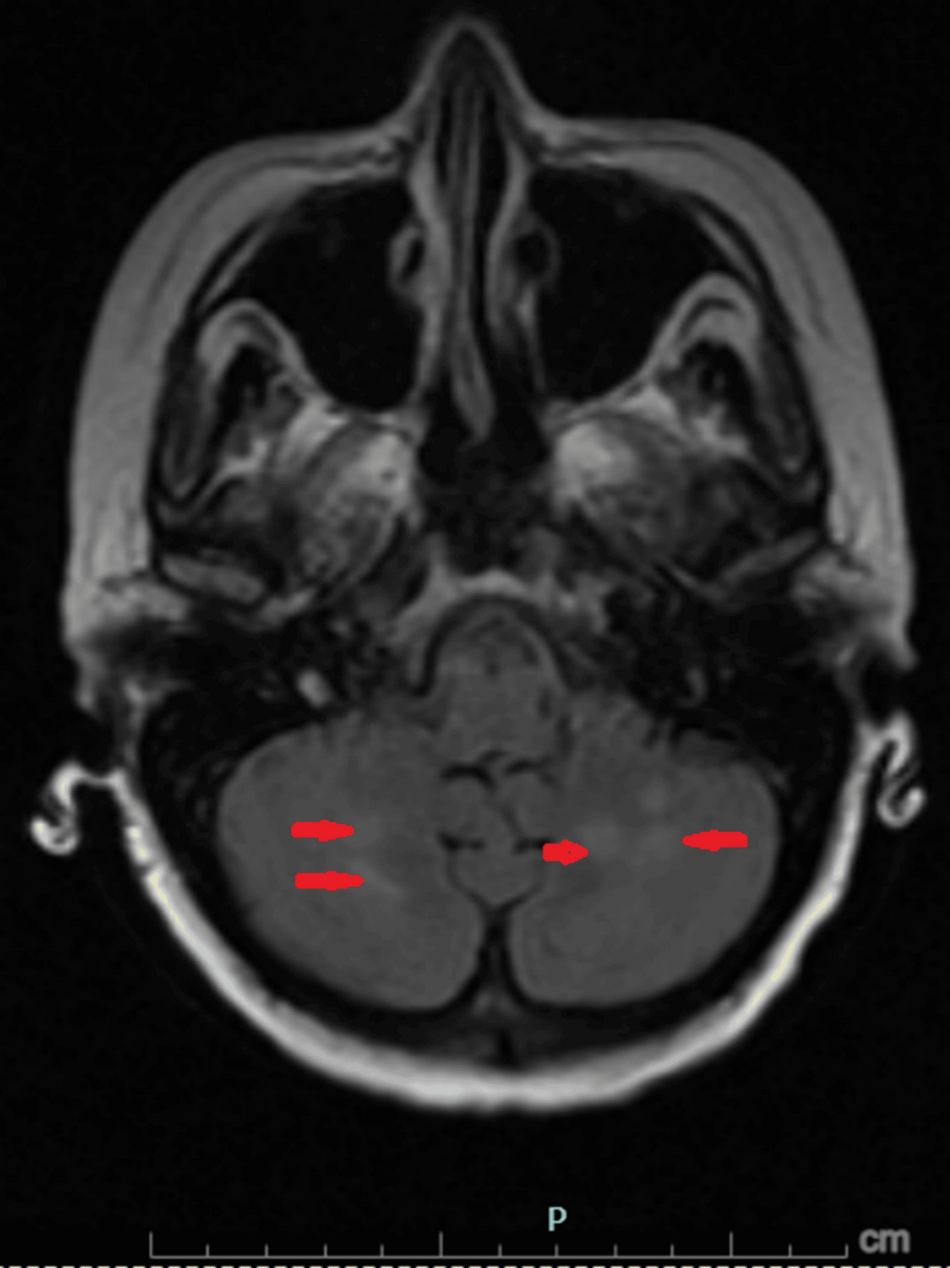
Cerebellar involvement in posterior reversible encephalopathy syndrome Fluid-attenuated inversion recovery (FLAIR) MRI images of the brain showing symmetric involvement of the bilateral cerebellar hemispheres (red arrows), consistent with posterior reversible encephalopathy syndrome in the appropriate clinical context.

Subsequent magnetic resonance imaging (MRI) of the brain with and without contrast (Figures 1-3) revealed symmetric subcortical fluid-attenuated inversion recovery (FLAIR) hyperintensities involving the bilateral cerebral and cerebellar hemispheres with posterior predominance, without diffusion restriction or abnormal enhancement. These findings were diagnostic of PRES.

Multidisciplinary evaluation concluded that the seizures were provoked in the setting of PRES, likely resulting from the combined effects of acute hypertension and cisplatin exposure. The patient was treated with levetiracetam, aggressive blood pressure control with antihypertensive therapy, and supportive care. Her mental status steadily improved, and no further seizures occurred. She was discharged in stable condition with outpatient neurology and oncology follow-up and counseling regarding seizure precautions and driving restrictions.

## Discussion

This case demonstrates PRES arising from the convergence of two well-recognized risk factors: hypertension and cisplatin-based chemotherapy [1,4,7]. PRES is increasingly viewed as a multifactorial process in which failure of cerebral autoregulation and endothelial dysfunction act synergistically to produce vasogenic edema, particularly within the posterior circulation [1,2].

Acute hypertension remains the most consistently identified precipitant of PRES, as abrupt elevations in blood pressure can overwhelm autoregulatory capacity, leading to hyperperfusion and endothelial injury [1,5]. Cisplatin has also been implicated in PRES through direct endothelial toxicity, oxidative stress, and disruption of the blood-brain barrier [1,6]. When present simultaneously, these insults may exert a cumulative effect, lowering the threshold for the development of PRES.

Oncology patients frequently present diagnostic challenges, as seizures and encephalopathy are often initially attributed to metastatic disease, metabolic disturbances, central nervous system infection, or medication effects. In this case, hepatic encephalopathy was considered early due to mild hyperammonemia and underlying hepatic metastasis. This was ultimately excluded based on preserved liver synthetic function and definitive neuroimaging findings.

Although infection was included in the initial differential diagnosis, the patient remained afebrile, blood cultures were negative, neuroimaging showed no features suggestive of infection, and symptoms improved without antibiotic therapy, effectively excluding an infectious cause. Brain metastasis was also excluded by MRI. This highlights the importance of maintaining a broad differential diagnosis and obtaining early MRI in new-onset seizures, especially if the initial CT scan is unremarkable.

Another component of the patient’s chemotherapy regimen, gemcitabine, has rarely been associated with PRES in isolated reports, especially when given in combination with cisplatin or carboplatin, suggesting potential synergistic endothelial toxicity [8]. The clinical presentation and imaging findings are typically reversible with discontinuation of the offending agent and supportive management, though outcomes depend on prompt recognition [6,7].

Treatment of PRES involves eliminating the precipitating cause, controlling blood pressure if elevated, seizure management, and supportive care. There is no specific therapy for PRES, and no randomized controlled trials have evaluated treatment interventions [2,5]. In our patient, blood pressure and seizure management, together with discontinuation of chemotherapy, were instituted.

PRES has a favorable prognosis, with most patients making a complete recovery when the precipitating cause is eliminated or treated [2,9]. Clinical symptoms and vasogenic brain edema typically resolve within a few days to a couple of weeks following treatment, particularly with prompt diagnosis and intervention [2,10]. However, PRES can result in irreversible changes, especially when intracranial hemorrhage or restricted diffusion are present. Epilepsy may develop in some cases [2,9]. In our patient, there was no recurrence of seizure or headache, and she returned to her baseline neurological status before discharge.

## Conclusions

PRES should be considered in oncology patients presenting with acute seizures and encephalopathy, particularly when acute hypertension and platinum-based chemotherapy coexist. This case underscores the importance of recognizing PRES in the setting of converging pathophysiologic insults. Early neuroimaging and prompt management can lead to favorable outcomes and neurologic recovery.
